# Defining and Detecting Malaria Epidemics in the Highlands of Western Kenya

**DOI:** 10.3201/eid0806.010310

**Published:** 2002-06

**Authors:** Simon I. Hay, Milka Simba, Millie Busolo, Abdisalan M. Noor, Helen L. Guyatt, Sam A. Ochola, Robert W. Snow

**Affiliations:** * University of Oxford, Oxford, United Kingdom; † Kenya Medical Research Institute/Wellcome Trust Collaborative Programme, Nairobi, Kenya; ‡ Ministry of Health, Nairobi, Kenya

**Keywords:** western Kenya, highlands, Plasmodium falciparum, malaria, epidemic, early detection

## Abstract

Epidemic detection algorithms are being increasingly recommended for malaria surveillance in sub-Saharan Africa. We present the results of applying three simple epidemic detection techniques to routinely collected longitudinal pediatric malaria admissions data from three health facilities in the highlands of western Kenya in the late 1980s and 1990s. The algorithms tested were chosen because they could be feasibly implemented at the health facility level in sub-Saharan Africa. Assumptions of these techniques about the normal distribution of admissions data and the confidence intervals used to define normal years were also investigated. All techniques identified two “epidemic” years in one of the sites. The untransformed Cullen method with standard confidence intervals detected the two “epidemic” years in the remaining two sites but also triggered many false alarms. The performance of these methods is discussed and comments made about their appropriateness for the highlands of western Kenya

Epidemics of all infectious diseases generate considerable public attention and are reported widely in the popular and scientific press. The definition of truly exceptional numbers of cases from commonly perceived “epidemics” is often difficult, however, particularly for widespread pathogens (*1*). *Plasmodium falciparum* malaria is extensive, prevalent, and increasing in sub-Saharan Africa (*2*–*4*). Stable endemic malaria predominates throughout the continent, but epidemics occur at the fringes of endemic areas, particularly among communities at the southernmost latitudes, across the arid regions of North Africa, and among the highlands of East, central, and Horn of Africa (*5*,*6*).

In the late 1980s and early 1990s, a series of malaria “epidemics” were reported in the western highlands of Kenya and other communities at high altitude in the subregion (*5*,*7*–*17*). A widely held view is that the transmission of *P. falciparum* in such communities is limited primarily by low ambient temperature and that small changes in temperature could therefore provide transiently suitable conditions for unstable transmission within populations that have acquired little functional immunity (*18*–*21*). Furthermore, the highlands of Kenya are densely populated and agriculturally productive. These factors have contributed to the Government of Kenya’s decision to define 15 districts in the western highlands (Figure 1.; [*22*]) as being prone to epidemics and thus meriting special attention for surveillance to increase epidemic preparedness (*23*).

**Figure 1:**
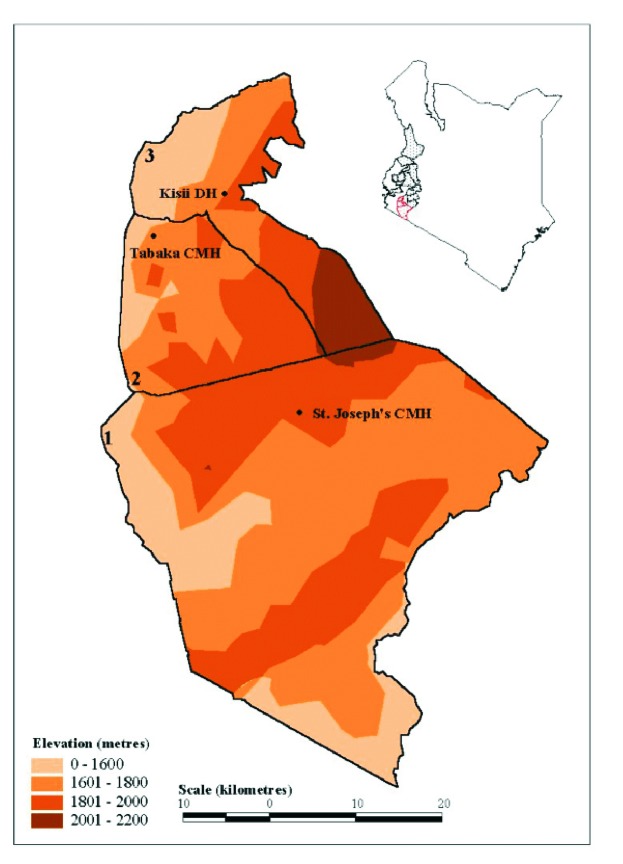
Locations of the three study hospitals and the administrative districts they serve in the highlands of western Kenya. The inset map of Kenya shows the 15 districts designated by the Government of Kenya as at risk from unstable, temperature-limited, and hence epidemic malaria. The three districts shaded in red are those in the large map. The St. Joseph’s Catholic Mission Hospital (CMH) at Kilgoris, Tabaka CMH, and Kisii District Hospital are shown within their administrative boundaries of (1) Trans Mara, (2) Gucha and (3) Kisii Central District, respectively. The districts are shown against a backdrop of a digital elevation model for which a key is provided. North is to the top of the page.

The World Health Organization’s (WHO’s) Roll Back Malaria’s efforts to manage epidemic malaria in sub-Saharan Africa include supporting the establishment of early detection (surveillance), early warning, and forecasting systems to provide adequate preparation time to prevent or contain malaria epidemics (*24**,**25*; URL: http://www.rbm.who.int/). The object of early detection (or epidemiologic surveillance) is to monitor a disease continually so that abnormal events can be identified rapidly, in the expectation that intervention efforts can be initiated in a timely manner (*26*,*27*). Extensive research on the optimization and comparison of surveillance algorithms exists (*28*–*34*); most published articles, however, are concerned with weekly reporting of rare infectious diseases in relatively wealthy countries. In technologically underdeveloped nations, governments have far fewer resources for disease prevention and medical care. Resource constraints in the health sector are often so severe that the time a health service employee may devote to surveillance will inevitably result in compromises elsewhere. In such circumstances, these cost-benefit considerations favor simple, robust surveillance systems (*35*).

We examined three simple techniques proposed for malaria epidemic detection (*24*) to evaluate what early warning information would have been provided if surveillance had been implemented using standard admissions records at three hospitals in the western Kenyan highlands during the late 1980s and 1990s. We did not explore the meteorologic correlates of temporal changes in malaria cases at these sites as a basis for malaria early warning (*6*,*36*–*38*), although this is the subject of ongoing research (*39*,*40*).

## Methods

### Study Area

Three hospitals providing inpatient clinical care were identified in the western Kenyan highlands (Figure 1). These hospitals were selected because malaria epidemics had been reported within the last 5 years where they were located, and complete clinical records, spanning more than 10 years, were available for review. The three hospitals were St Joseph’s Catholic Mission Hospital at Kilgoris in Trans Mara District (latitude 1.068 S, longitude 34.958 E; altitude 1,683 m); Tabaka Catholic Mission Hospital (latitude 0.751 S, longitude 34.663 E; altitude 1,684 m) in Gucha District; and Kisii District Hospital (latitude 0.684 S, longitude 34.770 E; altitude 1,815 m) in Kisii Central District. The hospitals serve varying catchment populations and are within 40 km of one another.

Each facility is located above 1,600 m, an altitude above that defined as characterizing highland/epidemic-prone malaria (*18*–*20*), although such limits have been challenged (*5*) The average altitudinal limits of the wider area shown in Figure 1 range from 1,600 to 2,200 m.

**Figure 2:**
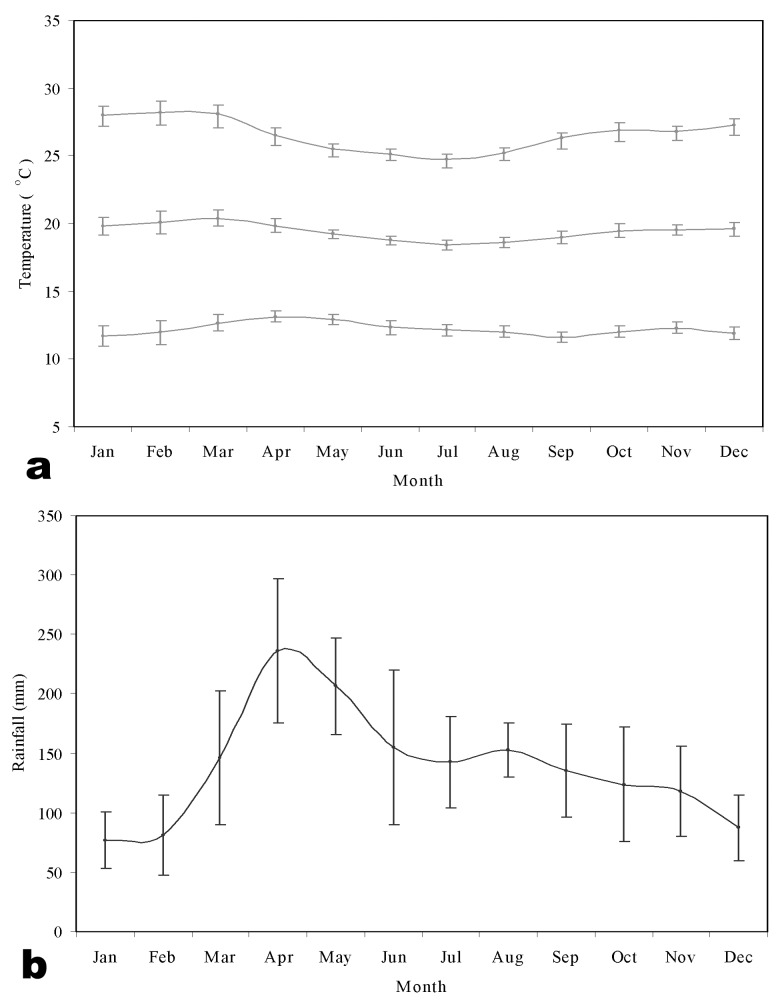
Meteorologic time-series for study hospitals. Temperature and rainfall profiles for a synoptic (1980-1995). (a) minimum (bottom), mean (middle), and maximum (top) monthly temperatures (°C); (b) average total monthly rainfall (mm). The error bars denote standard errors of the calculated month means.

Monthly temperature and rainfall data were extracted for January 1980 to December 1995 from an interpolated global climate surface at 0.5 x 0.5° spatial resolution (*41*,*42*), using georeferencing details from Tabaka Catholic Mission Hospital. The synoptic year (1980-1995) shows a remarkably stable mean monthly temperature of approximately 20°C (Figure 2a), with peak rainfall (approximately 200 mm) occurring in the months of April and May (Figure 2b), usually referred to as the “long rains.”

### Clinical Data

Hospital admission registers for every ward at each facility were located and sequentially reviewed to identify patient age, date, and cause of admission. Month- and age-tallied cases of “clinical malaria” were compiled for each complete year. Criteria used to select malaria cases were based on whether malaria was made as a primary, co-primary, or coincidental diagnosis by the admitting physician. Not all diagnoses were microscopically confirmed, and discharge diagnoses may have been different from those defined on admission, following further clinical and laboratory investigations. Nevertheless, patients at each facility were treated for malaria during the initial 24 hours of admission and represent the monthly clinical commitment to malaria case management at each hospital. Such data are used routinely to define epidemics by local health authorities and serve as the basis for increasing demands for resources.

In these analyses we consider only the pediatric malaria admissions (patients <15 years of age), who constituted approximately two thirds of the patients at each facility (Kilgoris, 14,079 adults and 30,793 children; Kisii, 44,043 adults and 84,648 children; and Tabaka, 23,692 adults and 55,871 children during the study period). The rationale is that children are more likely to give an accurate picture of local malaria transmission than adults, as they are less likely to have functional immunity or to have traveled and acquired the disease elsewhere. Cumulative monthly cases were also computed for each year to show the overall annual burden and acute, seasonal rises in malaria admissions. The years of exceptional malaria cases were defined simply as the 2 years of highest cases during the surveillance period.

### Epidemic Detection Techniques

We assumed a minimum set of requirements for resource-constrained, district-level health services in Kenya: access to a computer, limited knowledge of a spreadsheet application, and availability of at least 5 years of admission records from a health facility. For this reason, we focused on a subset of those techniques advocated by WHO for application to malaria surveillance in resource-constrained environments (*24*).

Epidemic alerts can be based on simple incidence thresholds only, as is common with meningococcal meningitis at the district level in sub-Saharan Africa (*43*–*46*); when a threshold is exceeded, an alert is triggered. The value of the threshold is usually determined from expert opinion informed by an examination of retrospective case data over wide geographic areas. This technique is not applicable to a single facility where accurate population denominator data (necessary to calculate incidence) are often not available and therefore not considered further.

Many epidemic surveillance techniques aim to identify points in a disease time-series outside the 95% confidence intervals of a normal distribution determined from the history of cases at that location. A method proposed by Cullen (*47*) uses the previous 5 years of data (in which epidemic years are arbitrarily excluded) to construct an admissions profile for an average year. The alert threshold for each month is then determined as the mean plus 2 times the standard deviation (strictly, the arithmetic mean plus 1.96 times the standard deviation should capture 95% of cases in normally distributed data [*48*]). This technique was successfully applied to cases of *Plasmodium vivax* malaria in northern Thailand during the 1980s (*47*). It has also been used for surveillance of *P. falciparum* malaria in the Madagascan highlands (*49*).

WHO has advocated the use of a conceptually similar method that triggers an alert when current cases exceed the upper 3rd quartile or the “upper normal limit” determined from 5 years of retrospective monthly case data (*50*). For 5 years of observations, quartile 0 is the minimum, quartile 1 the second lowest, quartile 2 the median, quartile 3 the second highest, and quartile 4 the maximum value of the series for any given month. If the current month’s cases exceed quartile 3, an alert is triggered. This method has been implemented to detect highland malaria epidemics in Ethiopia (*22*).

The Centers for Disease Control and Prevention has developed a further cumulative sum (c-sum) method for detecting epidemics. It is based on the construction of an average or base year, determined by calculating the expected number of cases using the average for that month (and the previous and following month) during the past 5 years (n=15) (*29*,*51*,*52*). For example, the expected number of cases for March 2000 would be derived from the average of February, March, and April admissions from 1995 to 1999, inclusive. A ratio of present to past cases is then usually presented as a current to past history graph (*53*), with values greater than one representing disease increases.

### Statistical Analysis

WHO, Cullen, and c-sum methods were tested on the series of pediatric malaria admissions data to evaluate their usefulness in the identification of epidemics, defined as the 2 years of highest numbers of cases. We modified the c-sum technique to provide 95% confidence intervals for the expected cases so that it could be evaluated against the other techniques. For each method, the expected cases in a given month were defined by the previous 5 years of data and sequentially updated for each new observation year in the series. “Epidemic years” were not excluded from the base years, as no objective criteria have been offered to define years that are epidemic and excluding these years would increase the likelihood of detecting epidemics**.** A skewness statistic that measures the degree of asymmetry in a distribution around the mean (Microsoft Excel 2000, Seattle, WA) was also applied to the data to test assumptions of normality in the admissions data. Positive or negative values indicate an asymmetric tail extending towards more positive or more negative values, respectively. The Cullen and c-sum techniques were then repeated by using log_10_ transformed childhood admissions data to investigate potential problems with the techniques that assume normally distributed data. Confidence intervals were determined for the Cullen and c-sum techniques on untransformed and log_10_ normalized admissions data by using the mean + (2x standard deviation) as well as the mean + (t value at 0.05 confidence interval x standard error), as is recommended for small sample sizes (*48*).

## Results

**Figure 3:**
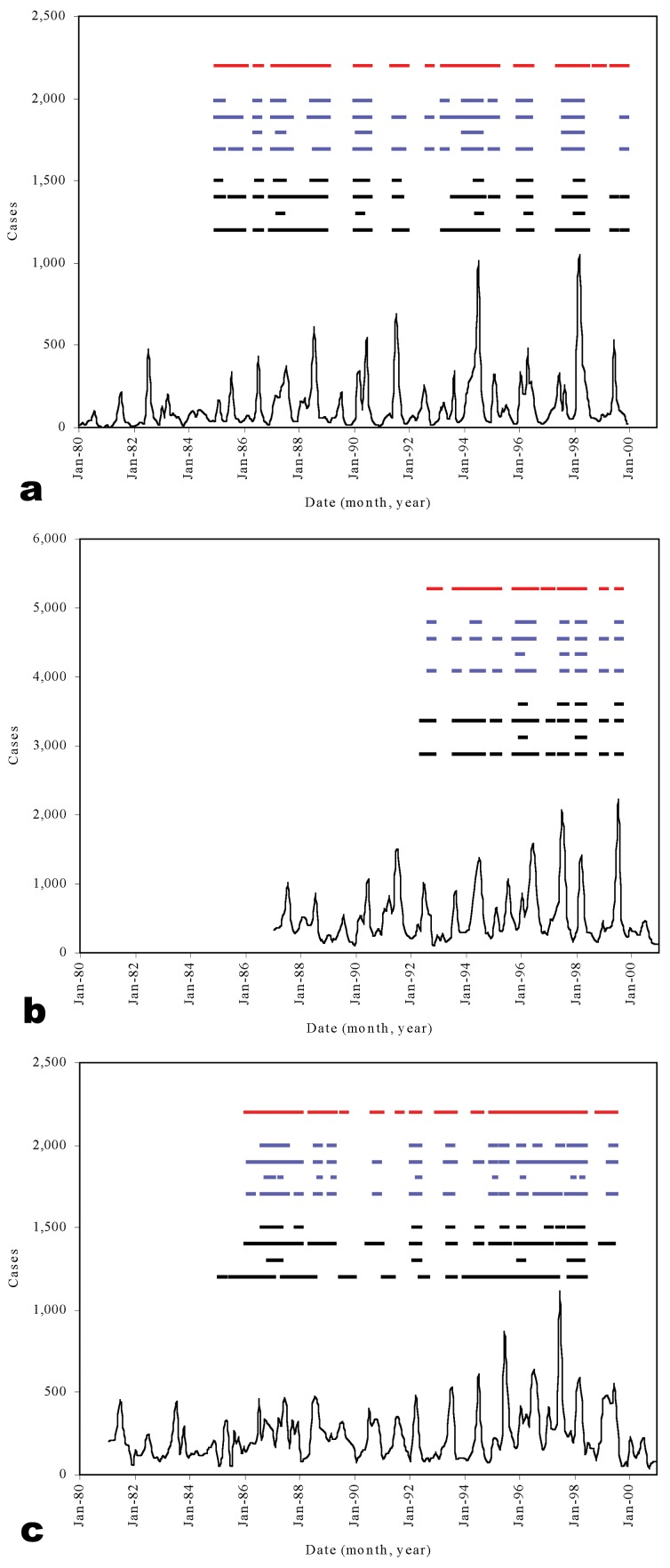
Time-series of child admissions and epidemic alerts for the three study hospitals.

**Figure 4:**
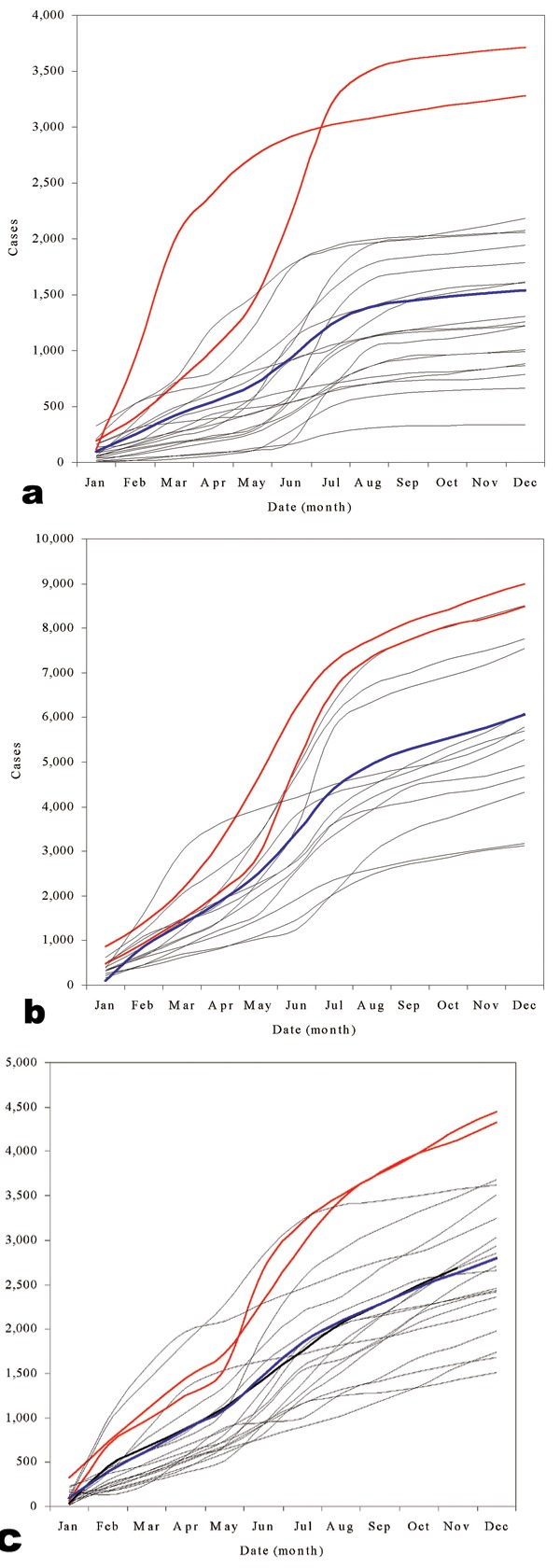
Cumulative case graphs for child admissions in the three hospitals. Cumulative child admissions (<15 years) in Kilgoris (a), Kisii (b), and Tabaka (c). All years for which data were available are shown, 1980-1999, 1987-2000, and 1981-2000 time periods for Kilgoris, Kisii and Tabaka, respectively. Black dashed lines are all “normal” years. The blue line shows mean average cumulative child admissions over all years. Red lines show epidemic years, defined as the 2 years of highest total admissions. For Kilgoris these exceptional years are 1994 and 1998, for Kisii they are 1996 and 1997, and for Tabaka they are 1997 and 1996.

Figure 3a-c shows pediatric admissions for the three study hospitals during the surveillance period. The graphs of cumulative cases (Figure 4a-c) show a distinct seasonality in admissions; the sharpest rise in case numbers occurred in June and July, immediately after the long rains in April and May (Figure 2b). The 2 years of highest case numbers were 1994 and 1998 for Kilgoris, 1996 and 1997 for Kisii, and 1997 and 1996 for Tabaka. In these so-called epidemic years, cases were often above normal in all months.

The child admissions data at each site were positively skewed with values of 2.88, 1.96, and 1.78 (skewness statistic = 0 for normal data series) for Kilgoris, Kisii, and Tabaka, respectively (Table 1). Log_10_ transformations of these data reduced the positive skew, thus helping normalize each series to values of -0.13, 0.34, and -0.08 for Kilgoris, Kisii, and Tabaka, respectively.

**Table 1:** Descriptive and skewness statistics for child admissions at three study hospitals, western Kenya

	Kilgoris (1980–1999)		Kisii (1987–2000)		Tabaka (1981–2000)	
Transformation	Normal	Log_10_	Normal	Log_10_	Normal	Log_10_
Mean	128.30	1.86	503.85	2.61	232.80	2.29
Minimum	3.00	0.48	95.00	1.98	35.00	1.54
Maximum	1,043.00	3.02	2,229.00	3.35	1,110.00	3.05
Sum	30,793.00	446.00	84,647.00	438.00	55,871.00	549.00
Count	240.00	240.00	168.00	168.00	240.00	240.00
Standard deviation	157.46	0.48	375.87	0.28	147.80	0.26
Standard error	10.16	0.03	29.00	0.02	9.54	0.02
Skewness	2.88	-0.13	1.96	0.34	1.78	-0.08

WHO methods concluded that 41.7%, 31.5%, and 42.8% of months in the surveillance period were epidemic for Kilgoris, Kisii, and Tabaka, respectively (Table 2; Figure 3a-c). The Cullen method showed fewer than half of these months to be epidemic, 14.4%, 10.2%, and 12.8%, respectively. The c-sum method indicated fewer still at 9.4%, 5.6%, and 10.6 %, respectively. Log_10_ transforming the child admissions data further reduced the proportion of months detected as epidemic. Adjusting the confidence intervals for small sample sizes had the opposite effect (Table 2). The WHO method and Cullen and c-sum techniques using the Kirkwood confidence intervals predicted approximately one third of all months during the surveillance period as epidemic (average 31.7%, range 14.8% to 42.8 %) (Table 2; Figure 3a-c). Strict statistical evaluation between the remaining techniques is difficult because of the problem of retrospectively determining what months were true epidemics; thus such evaluation was simply on the criteria of identifying the 2 years of highest cases (Figure 4). All techniques identified these 2 epidemic years in Kilgoris, but only the untransformed Cullen method with standard confidence intervals detected both epidemic years in Kisii and Tabaka as well.

**Table 2:** Comparison of total number of epidemic months detected by the World Health Organization (WHO), Cullen, and cumulative-sum techniques for three study hospitals, western Kenya^a^

Technique	Method	Kilgoris 1998-1999 N=180 (%)	Kisii 1992-2000 N=108 (%)	Tabaka 1986-2000 N=180 (%)
WHO	Not transformed	75 (41.7)	34 (31.5)	77 (42.8)
Cullen	Not transformed, SCI	26 (14.4)	11 (10.2)	23 (12.8)
	Not transformed, KCI	47 (26.1)	16 (14.8)	45 (25.0)
	Log_10_ transformed, SCI	13 (7.2)	4 (3.7)	12 (6.7)
	Log_10_ transformed, KCI	39 (21.7)	15 (13.9)	36 (20.0)
C-sum	Not transformed, SCI	17 (9.4)	6 (5.6)	19 (10.6)
	Not transformed, KCI	55 (30.6)	27 (25.0)	64 (35.6)
	Log_10_ transformed, SCI	6 (3.3)	3 (2.8)	8 (4.4)
	Log_10_ transformed, KCI	66 (36.7)	30 (27.8)	76 (42.2)

^a^Figures are number of months defined as epidemic in the monitoring period. Brackets are the percentage of the total months defined as epidemic.

## Discussion

Reports of epidemics in the highlands of western Kenya increased in frequency in the early 1990s (*10*,*12*,*54*,*55*); as a consequence, detection and control of epidemics became a priority for the recently launched national malaria strategic plan (*23*). This initiative forms part of a broader international effort to develop surveillance and warning systems for epidemic detection in Africa as part of the WHO Roll Back Malaria initiative (*24*,*56*). The definition of epidemics continues to confuse many public health practitioners specializing in common diseases such as malaria. Epidemics are more often defined in response to political necessity rather than by examining empirical data. Little critical examination of long-term clinical data against proposed methods for epidemic interpretation in nominally epidemic-prone areas of sub-Saharan Africa has occurred. To address this, we examined time series of pediatric malaria admission data during the late 1980s and 1990s from three hospitals located in districts of the western highlands of Kenya identified by the Ministry of Health as prone to epidemics.

Application of three primary epidemic detection methods indicated alert signals in most years of the test period with or without modifications. Rather than representing an inadequacy in the methods, this reflected the restricted utility of these approaches in areas of acutely seasonal malaria case burdens, characterized by a large degree of between-year variability in the timing of seasonal onset and a gradual increasing trend in admissions. Clearly, having such frequent epidemic alert signals makes the usefulness of such techniques in this particular area of the western Kenyan highlands questionable.

A further characteristic of this area is between-year variability in malaria incidence. During the 1990s, at least two important and dramatic seasonal rises in malaria occurred at each of the three hospitals (Figure 4). Sharp rises occurred during the months of February, and more commonly April or May (with the onset of the rains [Figure 2b]). Plotting monthly cumulative cases provided a more informative tool than traditional time-series plots to show seasonal deviations from previous years and simultaneously represented overall annual malaria cases. For the two exceptional years at each of the hospitals, the most sensitive of the “epidemic” detection methods shown in Figure 3 was the nontransformed Cullen technique that used standard confidence intervals. This technique, however, would also have given rise to a substantial number of false alarms during the observation period.

Applying the statistical techniques we have outlined highlights several methodologic issues that deserve comment, particularly for the Cullen and c-sum techniques, and should be considered by those advocating further application of these tools to common vector-borne diseases. First, mosquito-borne diseases that are sensitive to climate and hence are often seasonal, can show a skewed non-normal distribution in time. Methods that depend on arithmetic means and standard deviations (with their assumptions of data normality) to define alerts may require data transformation. Simple log_10_ transforms achieved data normalization and decreased the sensitivity of the techniques at all three facilities in this study. Second, each technique recommends using 5 years of retrospective admissions data so that standard deviations and hence alert thresholds for an average month are based on only five samples. A more appropriate formula for calculating the standard deviation in such situations has been proposed (*48*), although applying such modifications to these health facilities made the epidemic detection techniques substantially more sensitive. Third, when cases are increasing over the duration of the study, it is important to take a 5-year moving average to adjust the magnitude of the base year accordingly. Testing for the sensitivity of these techniques to the duration of moving average used was beyond the scope of this research but requires future investigation. Fourth, exclusion of “epidemic years” is an undefined procedure. For example, how many months detected as epidemic are needed in any year to prompt that year’s exclusion from the moving average, and after exclusion, what data are used to define the confidence intervals for alerts? This exercise demonstrates that many factors need to be more fully considered before widely advocating such techniques.

Our analyses used records of severe and complicated malaria admissions to tertiary-level health facilities, where diagnosis is often supported by microscopy. We have not applied the epidemiologic surveillance tools to patients with mild, ambulatory cases of malaria treated as outpatients. These latter data may provide a more robust tool for early detection, but they are also subject to imprecise clinical case definitions, where diagnosis is almost always made presumptively without microscopy. Improvements in the provision of microscopy in the diagnosis of outpatient malaria may facilitate improvements of these surveillance tools.

A further important problem that needs to be addressed is what constitutes an epidemic. Epidemic malaria was precisely described by MacDonald as “… an acute exacerbation of disease out of proportion to the normal to which the community is subject….Epidemics are common only in zones of unstable malaria, where very slight modification in any of the transmission factors may completely upset equilibrium and where the restraining influence of immunity may be negligible or absent, and they therefore show a very marked geographic distribution” (*57*,*58*).

The term epidemic is applied more liberally today for malaria in the Kenyan highlands; it is essentially used for any occurrence of cases in excess of normal. Much of the confusion around defining epidemics spatially or temporally relates to knowing what is (or should be) expected routinely. Endemic malaria, for example can show considerable expected temporal variation. This can relate to climate-driven variation, seasonality, interepidemic periods resulting from population dynamics, or long-term trends (*39*). These factors can all operate simultaneously and are not epidemics, although they may have substantial public health implications. Deviations from any of these expected variations are true epidemics if they result from a disturbance of the normal epidemiologic equilibrium (*50*). Such considerations are crucially important in the determination of the normal situation against which epidemics are measured.

The highlands of western Kenya is an area where so-called malaria epidemics have been increasingly reported. The area was recently highlighted by the government of Kenya as epidemic prone. Considerable international efforts are also being made to develop and promote early warning and improved case-detection systems for epidemic-prone areas (*24*,*56*,*59*). These results indicate that the simple epidemic detection techniques recommended to date require substantial refinement before they can be considered operationally robust, since they lack the required sensitivity in detecting aberrant case burdens. The further question as to whether these techniques are appropriate for facilities that have pronounced and acutely seasonal transmission of malaria is still open. The dual goals of technique development and a more comprehensive description of the local malaria epidemiology in this region are the subjects of ongoing research. A related article in this issue outlines the implications of these data for interpreting the epidemiology of *P. falciparum* malaria in this highland region of western Kenya (*60*).
